# Dynamic Modeling and Analysis of Flexible-Joint Robots with Clearance

**DOI:** 10.3390/s24134396

**Published:** 2024-07-06

**Authors:** Jing Wang, Shisheng Zhou, Jimei Wu, Jiajuan Qing, Tuo Kang, Mingyue Shao

**Affiliations:** 1 School of Mechanical and Precision Instrument Engineering, Xi’an University of Technology, Xi’an 710048, China; 1220210014@stu.xaut.edu.cn (J.W.); zhoushisheng@xaut.edu.cn (S.Z.); qingjiajuan@163.com (J.Q.); 1220210008@stu.xaut.edu.cn (T.K.); 2 Faculty of Printing, Packing and Digital Media Engineering, Xi’an University of Technology, Xi’an 710048, China; shaomingyue_xaut@163.com

**Keywords:** robot dynamics, clearance, flexible joint, modeling

## Abstract

The coupling effects of flexible joints and clearance on the dynamics of a robotic system were investigated. A numerical analysis was undertaken to reveal the coupling effects between flexible joints and clearance. The nonlinear spring-damping model and Coulomb model were applied to characterize the contact characteristics of the clearance, and a model for the flexible joint was formulated using the equivalent spring theory. An accurate robot model was established based on the clearance and joint flexibility characterization. The dynamic equation of a robot was obtained according to the Newton-Euler method. A comparative analysis was performed to assess the impacts of both the joint action of clearance and flexible joints and varying joint clearance values on the performance of the robot. The results showed that the coupling effects of flexible joints and clearance had a negative impact on the system dynamic performance. The amplitudes of the dynamic responses caused by the clearance are weakened by the flexible joint, but it leads to the lag of the system response. This study served as the theoretical foundation for exploring precise control techniques in robotics research.

## 1. Introduction

With the wide application of robots, the dynamic performance and control accuracy of robots are increasingly required, and developing a more precise dynamic model for robots is essential [1]. In conventional studies, the joints of robots are considered as ideal rigid joints [2]. The effects of flexibility and clearance have not been considered. However, the harmonic reducer is often used to improve the transmission efficiency in the actual robot structure, in which the flexible parts show torsional elasticity, causing the output angle of the motor to deviate from the actual rotation angle of the robot link and then increase the joint flexibility of the robot [3]. The existence of flexibility will change the response characteristics of the system and affect the prediction accuracy of the response to the whole structure. Therefore, the influence of flexible joints should be considered in the modeling process, and the joints cannot be simply set as rigid. In addition, due to factors such as manufacturing and assembly errors, clearances will inevitably occur in robot joints, which cannot be eliminated in precision manufacturing [4]. In an ideal condition, the journal of the joint and the bearing should have the same radius, but the radius of the journal and the bearing in the actual production will be different, resulting in clearance between the journal and the bearing, that is, the joint clearance. The joint clearance can lead to significant contact forces and unwanted vibration and noise, adversely affecting system dynamic behaviors. To predict the robot dynamics more accurately and improve the control accuracy of the robot, considering the flexible joints and clearances in the robot dynamic modeling and analysis is important.

Many scholars have studied the dynamic model of robots. Zhang and Wu [2] established the dynamic equation of a robot and studied the anti-interference control. Duan et al. [5] compared the dynamic and kinematic responses of the robot under different friction models and analyzed the interaction between various joints. Madsen et al. [6] considered the friction of the system in the dynamic modeling of a robot, and parameters in the model were identified, and the influence of load temperature and other factors on the characteristics of the robot was analyzed. Furthermore, there is a large amount of research on hybrid robots [7,8,9,10]. In the above research, the system is assumed to be rigid, and the influence of flexibility is ignored. Structural flexibility generally includes flexible links and joints in the analysis of multibody systems. Flexible links are links of a mechanism that have elastic deformation, which makes the system have flexible characteristics. Zhang and Yuan [11] examined the dynamic modeling and incorporated feedback control mechanisms for flexible-link robots. Ban et al. [12] investigated the influence of link flexibility on chaotic and bifurcation behaviors exhibited by robots. Based on the flexible-link model, Peza-Solis et al. [13] studied the trajectory tracking of the robot. The rigidity of the link in industrial robots is strong, and the length is short, so it is not necessary to consider the flexible link in modeling. However, the harmonic drive mechanism is generally used at the joint, and the harmonic rotation brings flexibility to the joint, which reduces the action accuracy of the system [14]. Spong [15] first proposed the torsion spring model to characterize the flexible joint and studied the feed-back linearization control of flexible-joint robots. Ruderman et al. [16,17,18,19] conducted a systematic study on flexible-joint robots and analyzed the problems of system modeling, control, and stability. Fateh [20] considered flexible joints in the investigation of the nonlinear control of robots. Spyrakos Papastavridis and Dai [21] also studied the control and trajectory tracking of flexible-joint robots. Farah et al. [22] explored vibration control strategies using a flexible-joint robot model, demonstrating that accounting for joint flexibility in the model enhanced the effectiveness of the implemented control measures. In addition, dynamic linearization analysis and modal studies of flexible-joint robots have been investigated by Do et al. [23]. Jing et al. [24] introduced a recursive approach for examining the inverse dynamics of robots with flexible joints, providing a basis for control optimization. Clearance is typical in mechanical systems, and there have been a lot of studies on clearance. Based on establishing the representation model, Flores et al. [25,26,27,28,29] investigated the kinematic properties of mechanisms incorporating clearance and verified its effectiveness through experiments. Wang et al. [30,31] formulated a contact force model for clearance. Gao et al. [32] examined the effects of clearance in a four-bar mechanism and utilized the coordinate partitioning technique to address the governing equation and found that friction reduces the extent of collision. Clearance as an uncertain factor increases the sensitivity of the system. Xiang and Yan [33] treated clearance as an uncertain parameter and quantified the influences on the dynamic behavior of space robots. Tang et al. [34] discussed the implications of uncertain clearance joints on the performance of robots and applied nonprobability theory to analyze the motion reliability of the system. Recently, Chen and Xu [35] modeled and simulated a driving robot with multiple clearances, evaluating its nonlinear response and affirming the model efficacy through rigorous performance evaluations. Wang et al. [36] delved into the three-dimensional representation of an assembly robot that accounts for clearance. Therefore, large clearance increases the motion amplitude of the robot and affects the work efficiency.

Flexible joints and clearances during robot operation cannot be ignored. In previous studies, the effects of flexible joints and clearances on system dynamics were studied, and the coupling between them has not been considered. In robot dynamic modeling, the joint action of flexible joints and clearances should be taken into account, and an improved dynamic model should be established to obtain more accurate dynamic output, which is the basis of accurate control. Therefore, the coupling action of flexible joint and clearance is fully considered. A model for a flexible-joint robot with clearance is formulated, from which the multi-degree-of-freedom dynamic equations are derived. Subsequently, numerical simulations are employed to ascertain the system responses. The characteristics of rigid systems without clearance, rigid systems with clearance, and flexible systems with clearance are compared and analyzed.

## 2. Modeling of Joint Clearance

**Figure 1:**
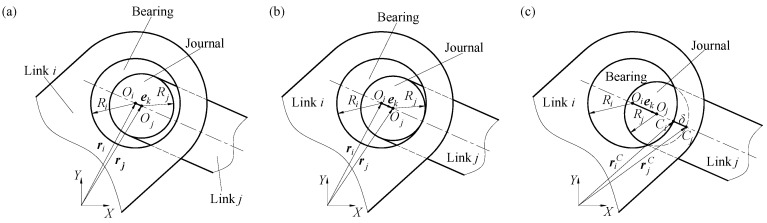
The mathematical model of clearance: (**a**) non-contact mode, (**b**) critical contact mode, (**c**) contact mode.

Figure 1 depicts a representation of the mathematical framework for modeling joint clearance. The center of bearing is $O_{i}$, the radius is $R_{i}$, the center of the journal is $O_{j}$, the radius is $R_{j}$, and the position vectors of the bearing and journal center in the global coordinate XY are $r_{i}$ and $r_{j}$, respectively. Radial clearance of the joint is $c=R_{i}-R_{j}$. Based on the varying relative positions of bearings and journals during motion, clearance joints are classified into three distinct categories: non-contact mode (Figure 1a), critical contact mode (Figure 1b), and contact mode (Figure 1c). In the continuous contact mode, the contact points on the bearing and journal are $C_{i}$ and $C_{j}$, respectively, and the contact point position vectors are $r_{i}^{C}$ and $r_{j}^{C}$ respectively.

The offset between the bearing and journal is referred to as the eccentric distance, which can be formulated as follows:

(1) $$e_{k}=r_{j}-r_{i}$$

The magnitude of the eccentric distance is as follows:

(2) $$e_{k}=\sqrt{e_{k}^{T}e_{k}}=\sqrt{e_{kx}^{2}+e_{ky}^{2}}$$

Then, the contact normal vector is as follows:

(3) $$n=\frac{e_{k}}{e_{k}}$$

According to the diagram of the contact model, the contact depth is as follows:

(4) $$\delta =e_{k}-c$$

According to the geometric relationship in Figure 1c, the expression can be formulated to depict the position and velocity of the point of contact as follows:

(5) $$r_{i}^{C}=r_{i}+R_{i}n\;\;\;r_{j}^{C}=r_{j}+R_{j}n$$

(6) $${\dot{r}}_{i}^{C}={\dot{r}}_{i}+R_{i}\dot{n}\;\;\;{\dot{r}}_{j}^{C}={\dot{r}}_{j}+R_{j}\dot{n}$$

Then, the normal collision velocity and tangential collision velocity are as follows:

(7) $$v_{n}={\left({\dot{r}}_{j}^{C}-{\dot{r}}_{i}^{C}\right)}^{T}n$$

(8) $$v_{t}={\left({\dot{r}}_{j}^{C}-{\dot{r}}_{i}^{C}\right)}^{T}t$$

where t is the tangential unit vector, which is perpendicular to the normal vector n.

**Figure 2:**
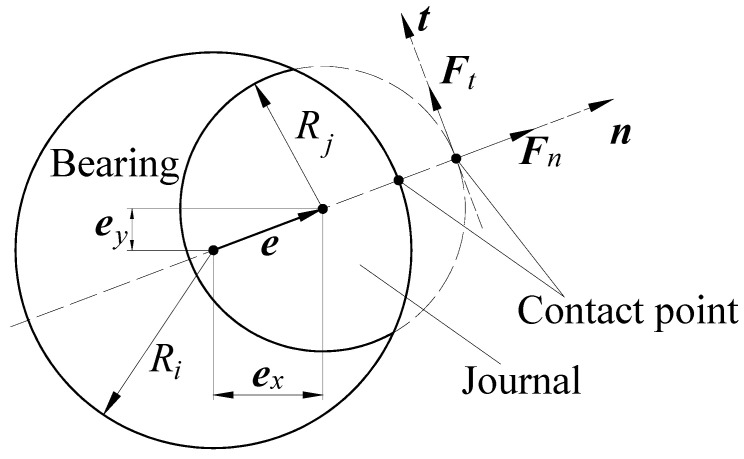
The tangential force and normal force during the collision process.

The tangential force $F_{t}$ and normal force $F_{n}$ during the collision process are shown in Figure 2. There has been some research on the characterization of contact forces during collision [25,26,27,37,38]. Hertz [39] laid the foundation for the characterization of elastic contact forces. However, the energy dissipation during collision is ignored in this study. Energy dissipation is considered in the study of Kelvin and Voigt, while the model cannot represent the nonlinearity and is only applicable to contacts with highimpact velocity [40]. The prevalent L-N model [41] captures contact nonlinearity, enabling its application for characterizing low-speed collisions.

Utilizing the L-N model, the normal contact force at the joint can be derived as follows:

(9) $$F_{n}=K\delta^{n}+\eta \delta^{n}\dot{\delta }$$

where K is generalized stiffness, n represents a nonlinear index that correlates with material properties, usually n=1.5 for metal materials [32,42]; the damping coefficient is η; and $\dot{\delta }$ is the relative contact speed.

According to the Coulomb model, the tangential force during collision is expressed as follows:

(10) $$F_{t}=-\mu F_{n}\mathrm{sgn}(v_{t})$$

where μ is the friction coefficient and sgn(·) is the sign function.

When contact occurs, the resultant force at the contact point is as follows:

(11) $$F_{c}=F_{n}+F_{t}$$

## 3. Modeling of the Flexible-Joint Robots with Clearance

**Figure 3:**
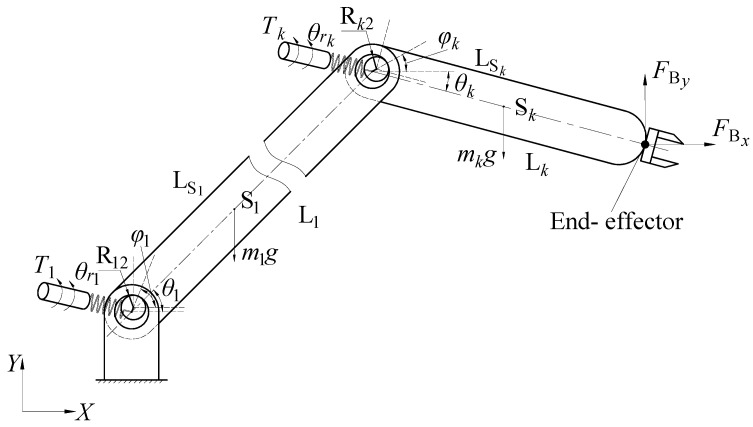
Mechanical model of flexible-joint robots with clearances.

The model of the flexible-joint robots with clearances is demonstrated in Figure 3. Each link is driven by an independent motor, and the torsion spring between the rotor of the motor and the link is given to characterize the reducer, which reflects the joint flexibility. The representation of joint clearance in the figure is an exaggeration. The joint angle $\theta_{i}$, the rotor angle $\theta_{ri}$, and the contact angle due to clearance $\varphi_{i}$ are the parameters considered in the modeling. The center of mass of the link is $S_{i}$; $F_{Bx}$ and $F_{By}$ are the components of the external force.

Common approaches for deriving dynamic equations in multi-body systems include the Lagrange method and the Newton–Euler method. In this paper, the influence of joint space is considered, and the internal action between components should be analyzed, so it is more appropriate to establish the robot dynamic equation using the Newton–Euler method. For a multi-link system with clearances, the centroid position of the link k (k=1,2,…) is formulated as follows:

(12) $$\left[\begin{array}{c} x_{S_{k}}\\ y_{S_{k}} \end{array}\right]=\left[\begin{array}{c} e_{1x}\\ e_{1y} \end{array}\right]+\cdots +L_{k-1}\left[\begin{array}{c} \cos\theta_{k-1}\\ \sin\theta_{k-1} \end{array}\right]+L_{S_{k}}\left[\begin{array}{c} \cos\theta_{k}\\ \sin\theta_{k} \end{array}\right]+\left[\begin{array}{c} e_{kx}\\ e_{ky} \end{array}\right]$$

where $e_{kx}$ and $e_{ky}$ are the components of the eccentricity. $e_{1x}$ and $e_{1y}$ are the components of the eccentric distance $e_{1}$ in *X* and *Y*, respectively. $L_{k-1}$ is the length of link k−1, and $L_{S_{k}}$ is the length between the center of gravity and the end of link k.

Then, the velocity and acceleration of the center of mass of the connecting rod is derived as follows:

(13) $$\left[\begin{array}{c} {\dot{x}}_{S_{k}}\\ {\dot{y}}_{S_{k}} \end{array}\right]=\left[\begin{array}{c} {\dot{e}}_{1x}\\ {\dot{e}}_{1y} \end{array}\right]+\cdots +L_{k-1}{\dot{\theta }}_{k-1}\left[\begin{array}{c} -\sin\theta_{k-1}\\ \cos\theta_{k-1} \end{array}\right]+L_{S_{k}}{\dot{\theta }}_{k}\left[\begin{array}{c} -\sin\theta_{k}\\ \cos\theta_{k} \end{array}\right]+\left[\begin{array}{c} {\dot{e}}_{kx}\\ {\dot{e}}_{ky} \end{array}\right]$$

(14) $$\begin{array}{l} \left[\begin{array}{c} {\ddot{x}}_{S_{k}}\\ {\ddot{y}}_{S_{k}} \end{array}\right]=\left[\begin{array}{c} {\ddot{e}}_{1x}\\ {\ddot{e}}_{1y} \end{array}\right]+\cdots +L_{k-1}{\ddot{\theta }}_{k-1}\left[\begin{array}{c} -\sin\theta_{k-1}\\ \cos\theta_{k-1} \end{array}\right]+L_{k-1}{\dot{\theta }}_{k-1}^{2}\left[\begin{array}{c} -\cos\theta_{k-1}\\ -\sin\theta_{k-1} \end{array}\right]\\ +L_{S_{k}}{\ddot{\theta }}_{k}\left[\begin{array}{c} -\sin\theta_{k}\\ \cos\theta_{k} \end{array}\right]+L_{S_{k}}{\dot{\theta }}_{k}^{2}\left[\begin{array}{c} -\cos\theta_{k}\\ -\sin\theta_{k} \end{array}\right]+\left[\begin{array}{c} {\ddot{e}}_{kx}\\ {\ddot{e}}_{ky} \end{array}\right] \end{array}$$

According to the different motion modes of the joints, the calculation of the contact force is as follows:

(15) $$\left[\begin{array}{c} F_{ckx}\\ F_{cky} \end{array}\right]=Q\left[\begin{array}{cc} \cos\varphi_{k} & \sin\varphi_{k}\\ \sin\varphi_{k} & -\cos\varphi_{k} \end{array}\right]\left[\begin{array}{c} F_{nk}\\ F_{tk} \end{array}\right]\;\;\;Q=\{\begin{array}{c} 1,\delta \geq 0\;\mathrm{contact}\;\\ 0,\delta <0\;\mathrm{free}\;\mathrm{flight} \end{array}$$

If the link k is an end link, the balance equation of the link can be written as follows:

(16) $$\left[\begin{array}{c} F_{Bx}\\ F_{By} \end{array}\right]-\left[\begin{array}{c} F_{ckx}\\ F_{cky} \end{array}\right]-\left[\begin{array}{c} 0\\ m_{k}g \end{array}\right]=m_{k}\left[\begin{array}{c} {\ddot{x}}_{S_{k}}\\ {\ddot{y}}_{S_{k}} \end{array}\right]$$

From the Euler equation $N=J_{S}\dot{\omega }+\omega \times (J_{S}\omega )$, the moment equation of the end link is derived as follows:

(17) $$\begin{array}{l} K_{rk}(\theta_{rk}-\theta_{k})-F_{Bx}L_{k}\sin\theta_{k}+F_{By}L_{k}\cos\theta_{k}\\ +F_{ckx}R_{k2}\sin\varphi_{k}-F_{cky}R_{k2}\cos\varphi_{k}-m_{k}gL_{S_{k}}\cos\theta_{k}=J_{k}{\ddot{\theta }}_{k} \end{array}$$

where $K_{rk}$ is the stiffness of the torsional spring, $\theta_{rk}-\theta_{k}$ is the angular deformation between rotor and link, and $R_{k2}$ is the radius of the journal in the joint.

If the link k is not an end link, the equation is expressed as follows:

(18) $$\left[\begin{array}{c} F_{c(k+1)x}\\ F_{c(k+1)y} \end{array}\right]-\left[\begin{array}{c} F_{ckx}\\ F_{cky} \end{array}\right]-\left[\begin{array}{c} 0\\ m_{k}g \end{array}\right]=m_{k}\left[\begin{array}{c} {\ddot{x}}_{S_{k}}\\ {\ddot{y}}_{S_{k}} \end{array}\right]$$

(19) $$\begin{array}{l} K_{rk}(\theta_{rk}-\theta_{k})-F_{c(k+1)x}(L_{k}\sin\theta_{k}+e_{(k+1)y}+R_{(k+1)2}\sin\varphi_{k+1})\\ +F_{c(k+1)y}(L_{k}\cos\theta_{k}+e_{(k+1)x}+R_{(k+1)2}\cos\varphi_{k+1})\\ +F_{ckx}R_{k2}\sin\varphi_{k}-F_{cky}R_{k2}\cos\varphi_{k}-m_{k}gL_{S_{k}}\cos\theta_{k}=J_{k}{\ddot{\theta }}_{k} \end{array}$$

The equation of rotor k in a flexible-joint robot is established as follows:

(20) $$J_{rk}N_{k}^{2}{\ddot{\theta }}_{rk}+K_{rk}(\theta_{rk}-\theta_{k})=T_{k}$$

where $J_{rk}$ is the rotational inertia of the rotor, $N_{k}$ is the deceleration ratio of the joint, and $T_{k}$ is the driving torque generated by the motor.

**Figure 4:**
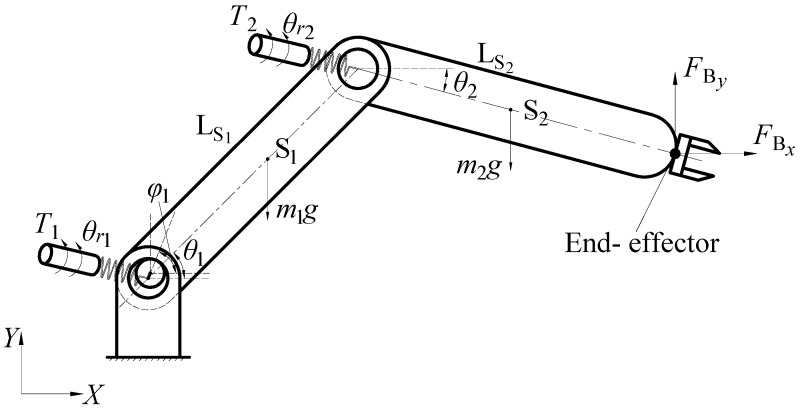
Simplified model of flexible-joint robot with clearance.

In this paper, the two-link flexible-joint robot with clearance is analyzed. Figure 4 presents the simplified model of the system.

Utilizing Equations (14)–(18), one can derive the dynamic equations for a multi-degree-of-freedom flexible-joint robot with joint clearance as follows:

(21) $$\left\{ \begin{array}{l} F_{2x}-F_{c1x}=m_{1}{\ddot{x}}_{S_{1}}\\ F_{2y}-F_{c1y}-m_{1}g=m_{1}{\ddot{y}}_{S_{1}}\\ K_{r1}(\theta_{r1}-\theta_{1})-F_{2x}L_{1}\sin\theta_{1}+F_{2y}L_{1}\cos\theta_{1}\\ +F_{c1x}R_{12}\sin\varphi_{1}-F_{c1y}R_{12}\cos\varphi_{1}-m_{1}gL_{S_{1}}\cos\theta_{1}=J_{1}{\ddot{\theta }}_{1}\\ J_{r1}N_{1}^{2}{\ddot{\theta }}_{r1}+K_{r1}(\theta_{r1}-\theta_{1})=T_{1}\\ F_{Bx}-F_{2x}=m_{2}{\ddot{x}}_{S_{2}}\\ F_{By}-F_{2y}-m_{2}g=m_{2}{\ddot{y}}_{S_{2}}\\ K_{r2}(\theta_{r2}-\theta_{2})-F_{Bx}L_{2}\sin\theta_{2}+F_{By}L_{2}\cos\theta_{2}\\ -m_{2}gL_{S_{2}}\cos\theta_{2}=J_{2}{\ddot{\theta }}_{2}\\ J_{r2}N_{2}^{2}{\ddot{\theta }}_{r2}+K_{r2}(\theta_{r2}-\theta_{2})=T_{2} \end{array}\right.$$

## 4. Simulation and Analysis

In this paper, a two-link flexible-joint robot with clearance is investigated in parameter analysis, and the dynamic equation of the robot system is established. The dynamic equations are simulated in this section to analyze the influences of factors such as the presence of the flexible joint and clearance, and the influence of the size of the joint clearance on the dynamic characteristics of the robot.

### 4.1. Simulation Parameters and Model

**Figure 5:**
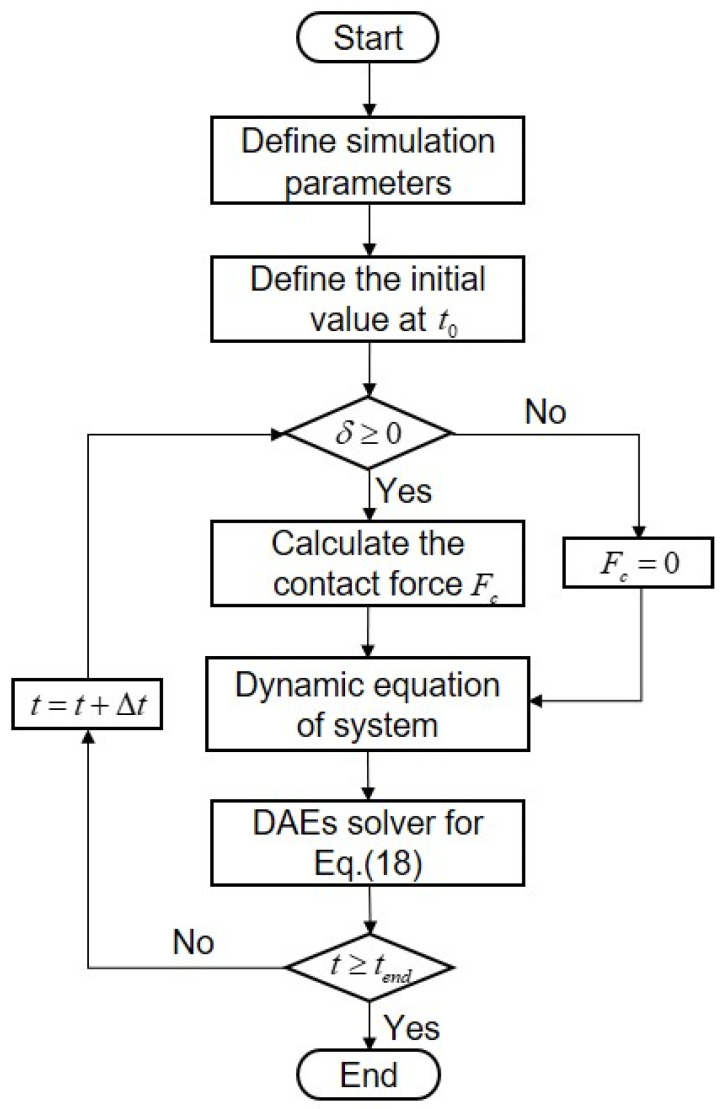
Flow chart of dynamic simulation.

The links of the robot studied in this paper are rigid, joint clearance exists at joint 1, and joints 1 and 2 are considered to be flexible joints. Therefore, the mathematical model of the robot contains six variables. In this paper, MATLAB is employed to simulate the robot model. The simulation parameters and the flow chart of the simulation are given in Table 1 and Figure 5, respectively. The specific simulation calculation flow of dynamic analysis of a flexible-joint robot with clearance is as follows:

(1) Define parameters required for simulation, set radius of journal $R_{12}=0.05$ m, size of clearance $c=5\times 10^{-5}$ m.
(2) Define the initial value of the system at $t_{0}$ and set the initial variables ${\left[e_{1x},e_{1y},\theta_{1},\theta_{2},\theta_{r1},\theta_{r2}\right]}_{t0}=[1\times 10^{-5},1\times 10^{-5},0,0,0,0]$.
(3) Calculate the contact depth according to the parameters and the variable values to further judge the contact state.
(4) Establish the contact model of the clearance and calculate the contact force $F_{c}$.
(5) Establish the system dynamic equation considering joint clearance and joint flexibility.
(6) Use DAEs solver to solve the system equation to obtain the variable values, and set the solver parameters: integral step is 1 × 10^−3^ and integral tolerance is 1 × 10^−7^.
(7) Repeat steps (3)–(6) until the simulation is over.

### 4.2. Influences of Flexible Joint and Clearance

**Figure 6:**
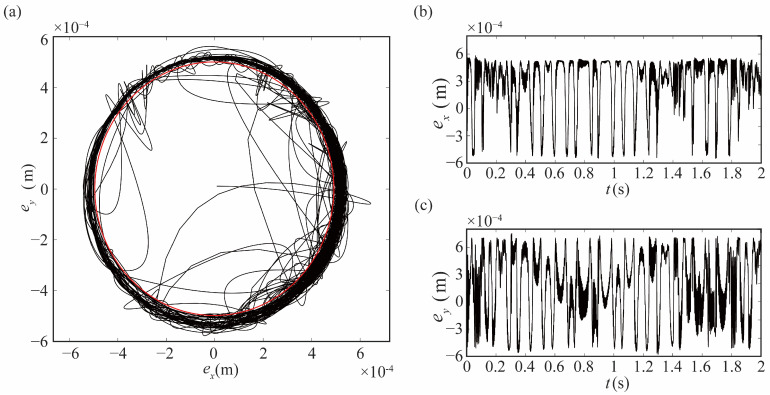
Locus of journal center of rigid joint: (**a**) locus of center, (**b**) locus in X direction with time, (**c**) locus in Y direction with time.

The dynamic behaviors of robots under different model parameters are explored in this research, and the main models are as follows: rigid-joint robot without clearance model (rigid-ideal, R-I), rigid-joint robot with clearance model (rigid-clearance, R-C), and flexible-joint robot with clearance model (flexible-clearance, F-C). The locus of the center of joint 1 is drawn in Figure 6a. The red line in the figure is the clearance circle, and the black is the locus of the center. Figure 6b,c show the motion locus in the X and Y direction, respectively. As seen from Figure 6, the motion mode between the bearing and the journal differs at different times. The locus outside the clearance circle indicates the continuous contact mode.

**Figure 7:**
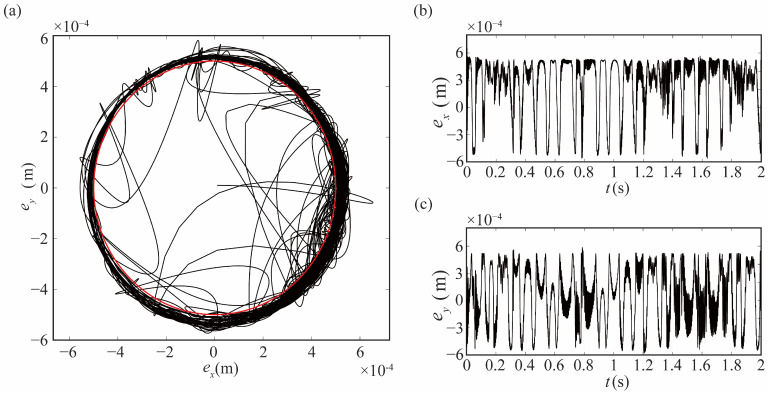
Locus of journal center of flexible joint: (**a**) locus of center, (**b**) locus in X direction with time, (**c**) locus in Y direction with time.

As shown in Figure 7, the locus of center of joint 1 and its time-varying trajectories in the X and Y directions are demonstrated when flexible joints are taken into account. Compared with Figure 6, it can be observed that the flexible characteristics of the joint affect the contact state, and the non-contact and impact increase.

**Figure 8:**
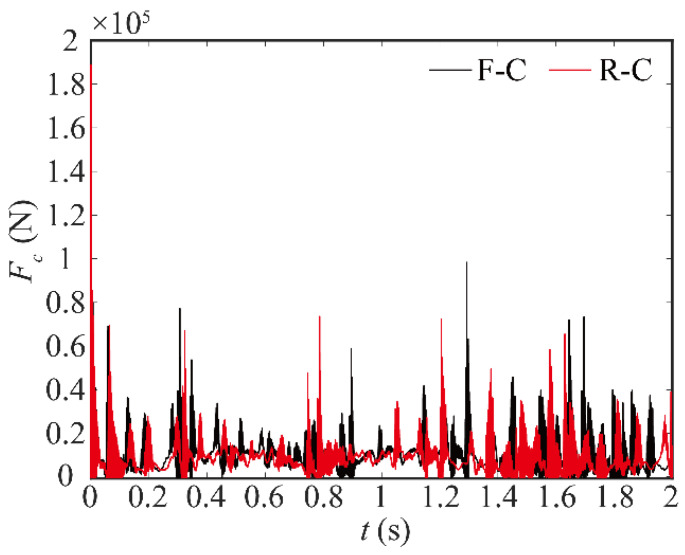
Contact force in joints.

The curve of contact-force change at robot joint 1 over time is given in Figure 8. The black line depicts the variation in the contact force of a rigid-joint robot with clearance, while the red line depicts the variation in a robot with the flexible joint and clearance. Within the clearance, a significant contact force is observed, exhibiting a pronounced pulse-like characteristic. The flexibility of joints increases the frequency of contact force, but the values decrease. This indicates that the flexible joint affects the characteristics of the joint clearance. There is a coupling between the flexible joint and the clearance.

**Figure 9:**
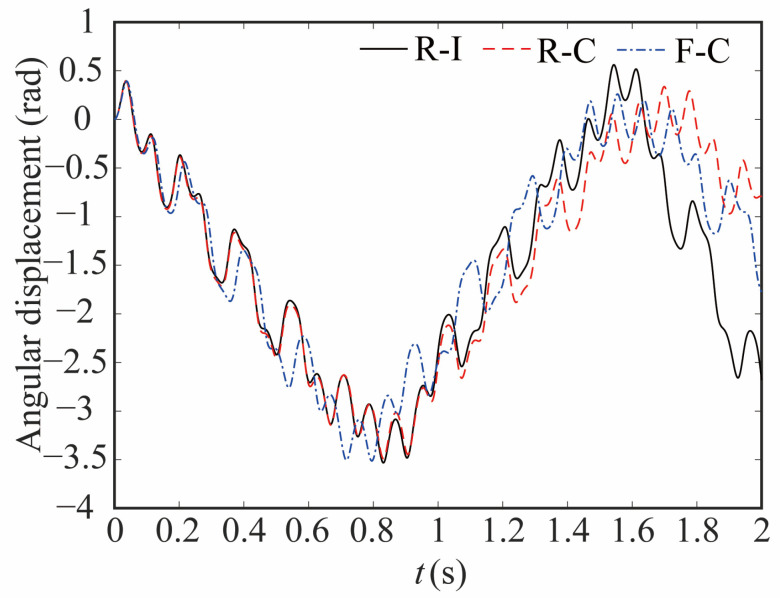
Comparative analysis of the angular displacement at joint 1.

**Figure 10:**
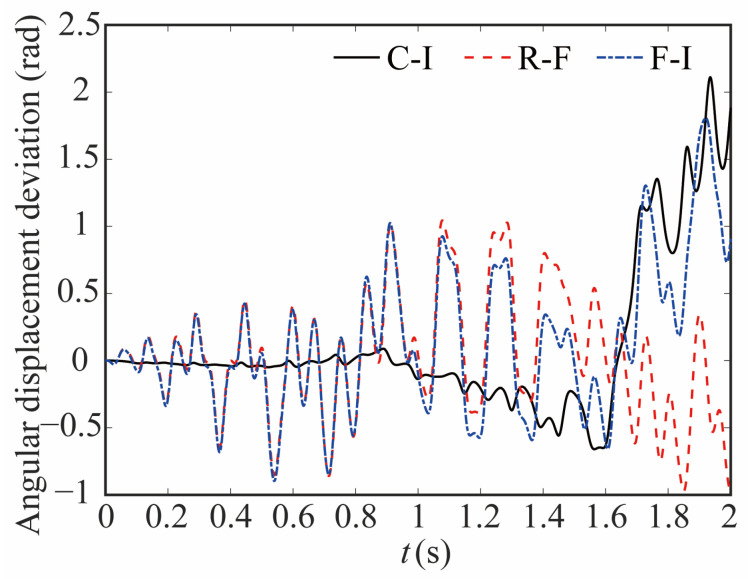
Deviation in angular displacement at joint 1.

The comparison of the angular displacement of joint 1 for different robot models is shown in Figure 9. The deviation between different models is given in Figure 10. The black-solid line represents the deviation of angular displacement between the rigid-clearance model and the rigid-ideal model (C-I), the red-dashed line represents the difference between the rigid-clearance model and the flexible-clearance model (R-F), and the blue-dotted line represents the deviation between the flexible-clearance model and the rigid-ideal model (F-I). It can be clearly observed that the angular displacement of joint 1 is large when considering the clearance, and the deviation increases gradually with time. When considering flexible joints, the angular displacement of joint 1 also increases. The angular displacement of joint 1 is larger than that of the ideal model when the clearance and flexible joint are considered at the same time, but the angular displacement deviation at the same time is smaller than that of the rigid-clearance model. This is because the deviation caused by flexible joints neutralizes some caused by clearance.

**Figure 11:**
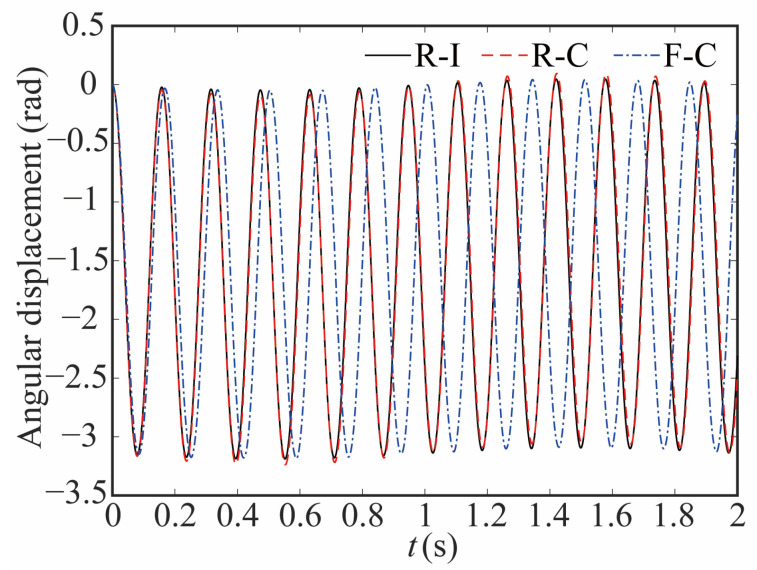
Comparative analysis of the angular displacement at joint 2.

**Figure 12:**
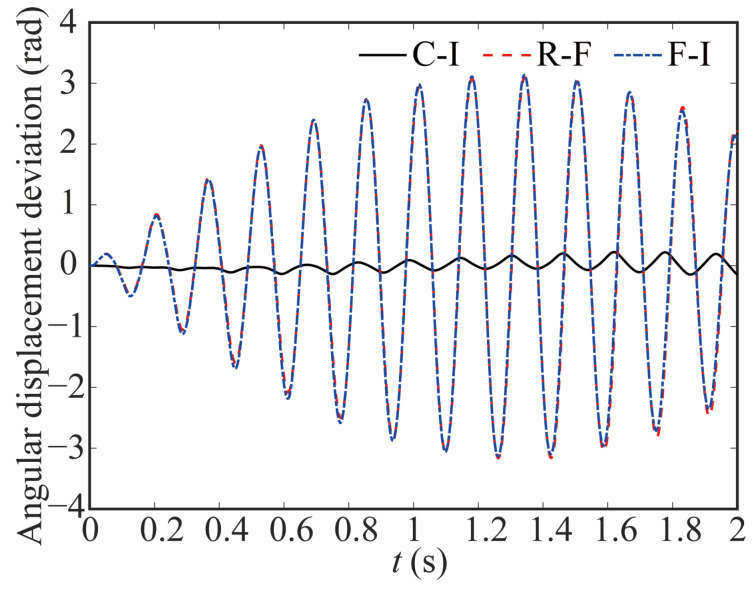
Deviation in angular displacement at joint 2.

Figure 11 and Figure 12 show the comparison of angular displacement for joint 2 and the deviation between different models, respectively. The clearance does not cause much change about angle of joint 2. However, when considering the influence of joint flexibility, the angle of joint 2 changes greatly. The angular displacement of the flexible joint lags notably in comparison to that of the rigid joint.

**Figure 13:**
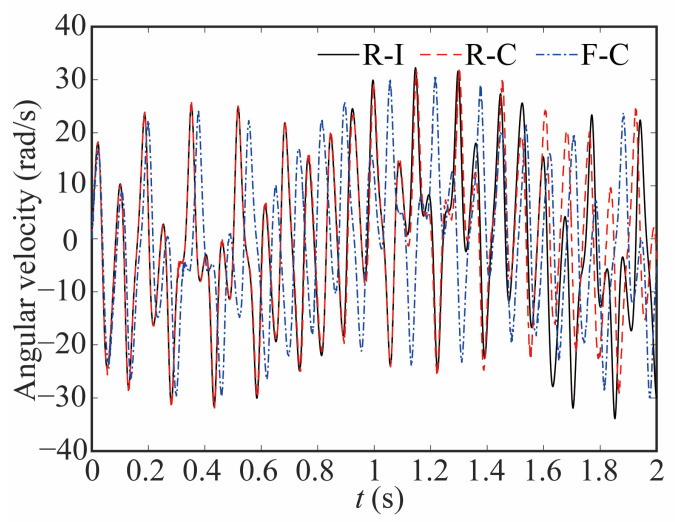
Comparative analysis of the angular velocity at joint 1.

**Figure 14:**
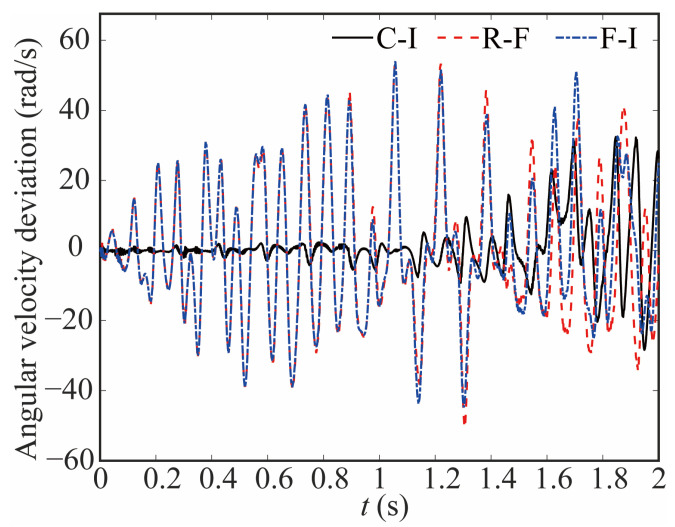
Deviation in angular velocity at joint 1.

Figure 13 and Figure 14 illustrate the variations in angular velocity at joint 1 for various robot models, highlighting the discrepancies among them. The angular velocity is larger in the flexible-clearance model, and the lag phenomenon is also obvious. Taking into account the flexible joint and clearance, the angular velocity curve of the joint lags significantly and exhibits a diminished amplitude when compared to that of the rigid-ideal model.

**Figure 15:**
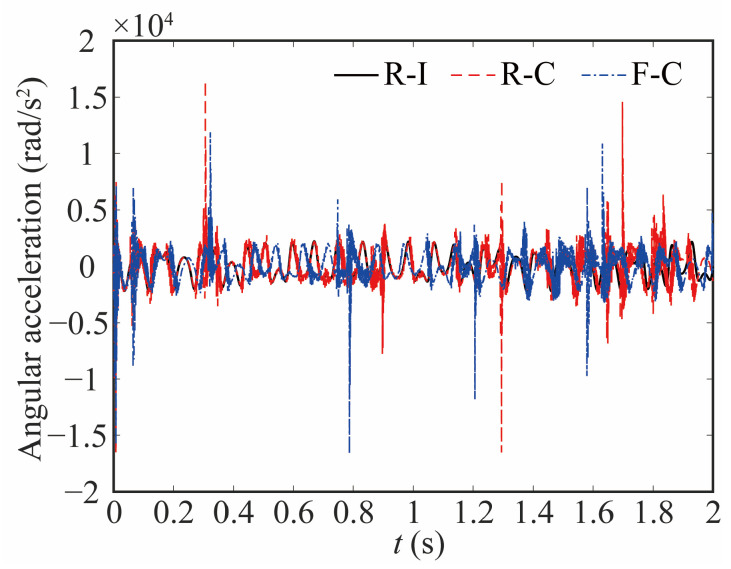
Comparative analysis of the angular acceleration at joint 1.

**Figure 16:**
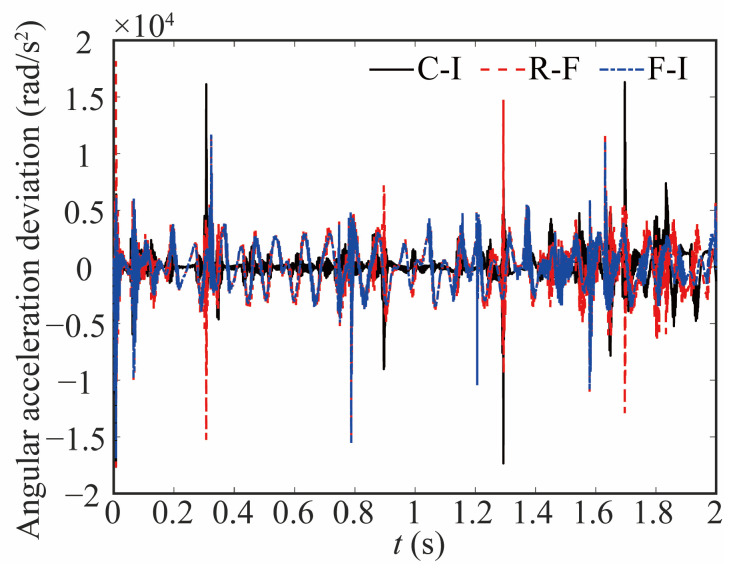
Deviation in angular acceleration at joint 1.

Figure 15 and Figure 16 show the change in the angular acceleration and the deviation, respectively. As seen from Figure 15 and Figure 16, the angular acceleration curve of joint 1 for the rigid-ideal model is relatively smooth. When the influence of clearance is taken into account, the amplitude of angular acceleration increases, and the curve shows impact peaks. The appearance of a peak means that there is a large contact force, which affects the stability of the robot. The value of the peak is relatively reduced when the flexible joint is considered. These things considered, the curve has a significant difference and noticeable lag when the flexible joint and clearance are coupled.

**Figure 17:**
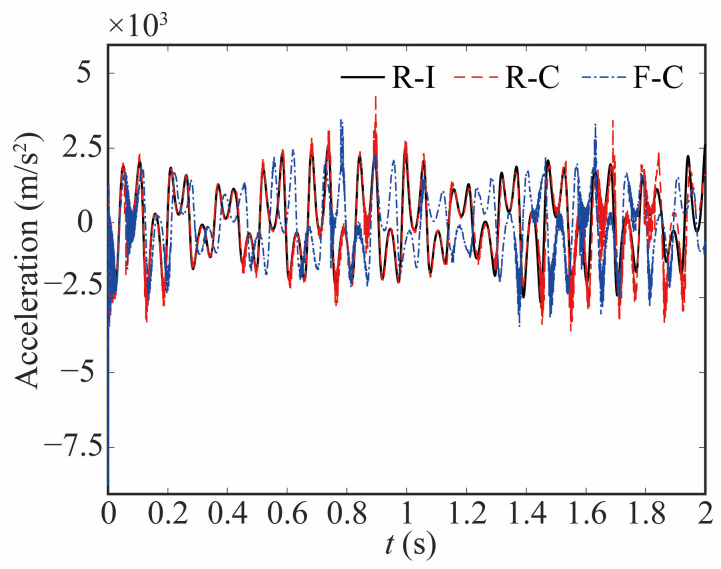
Comparative analysis of acceleration at end-effector in X direction.

**Figure 18:**
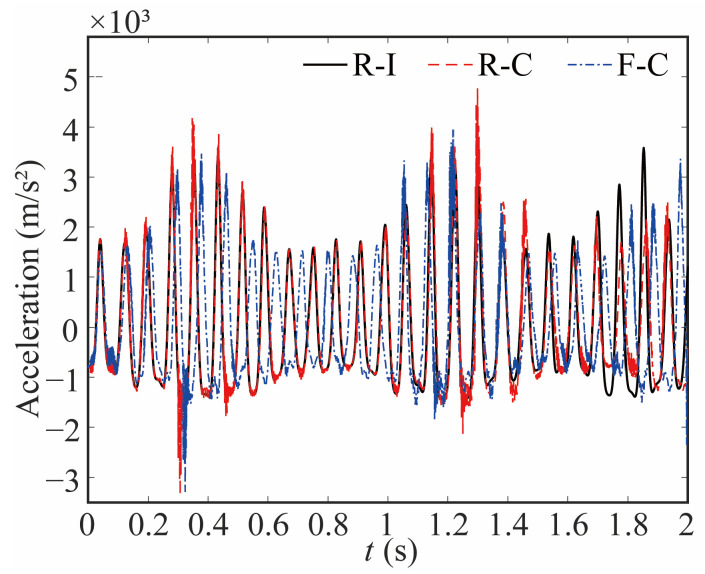
Comparative analysis of acceleration at end-effector in Y direction.

Flexible joints and clearance of the robot not only affect the motion of the joint but also greatly influence the dynamic characteristics of the end-effector. In Figure 17 and Figure 18, the acceleration curves of the end-effector are depicted. Clearance and flexibility exert a great influence on the acceleration of the end-effector. When clearance is taken into account, peaks and an augmentation in acceleration emerge, resulting in a considerable inertial force exerted on the robot and subsequently affecting its stability and accuracy.

### 4.3. Influences of Clearance Size

Clearance and flexible joints exert a notable impact on the robot performance. A detailed numerical analysis further reveals the influences of clearance size, coupled with the joint clearance and flexibility, on the robot functionality.

**Figure 19:**
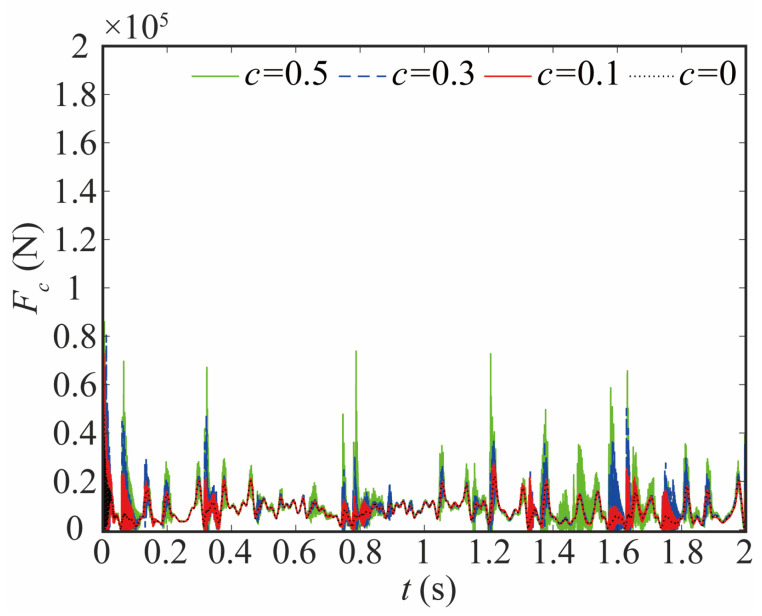
Comparison of contact force with different clearance sizes.

As depicted in Figure 19, the contact force at the joint exhibits a general upward trend, indicating an overall increase. Especially, the contact force at the peak increases significantly, as shown in Table 2. When *c* = 0.5, the frequency of the contact force at 1.41–1.72 s increases significantly. This indicates a rapid change in the motion state of the bearing and journal, which will decrease the stability of the system.

**Figure 20:**
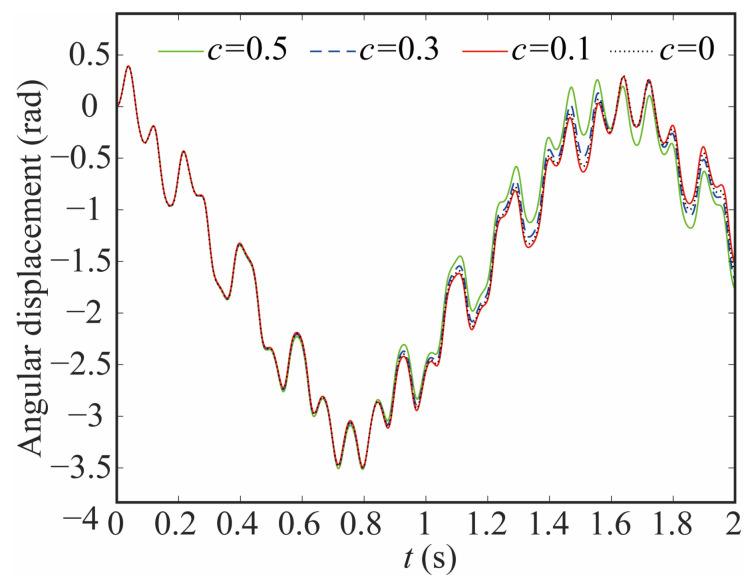
Angular displacement of joint 1 with different clearance sizes.

**Figure 21:**
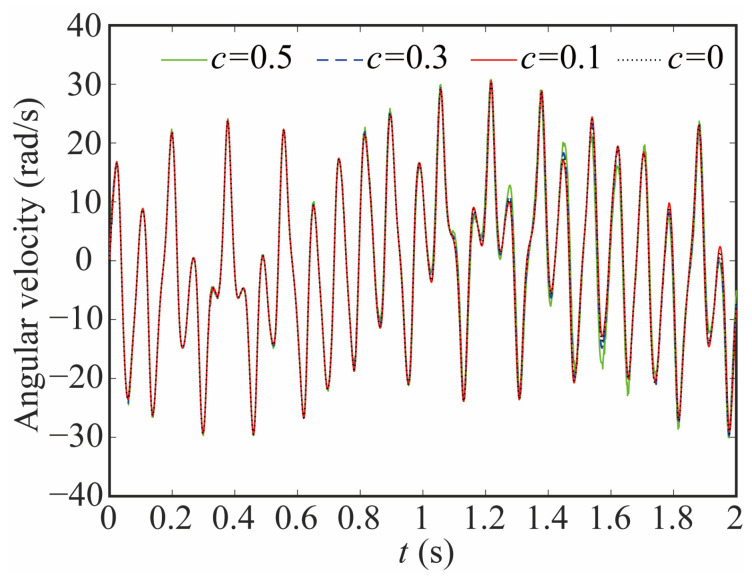
Angular velocity of joint 1 with different clearance sizes.

**Figure 22:**
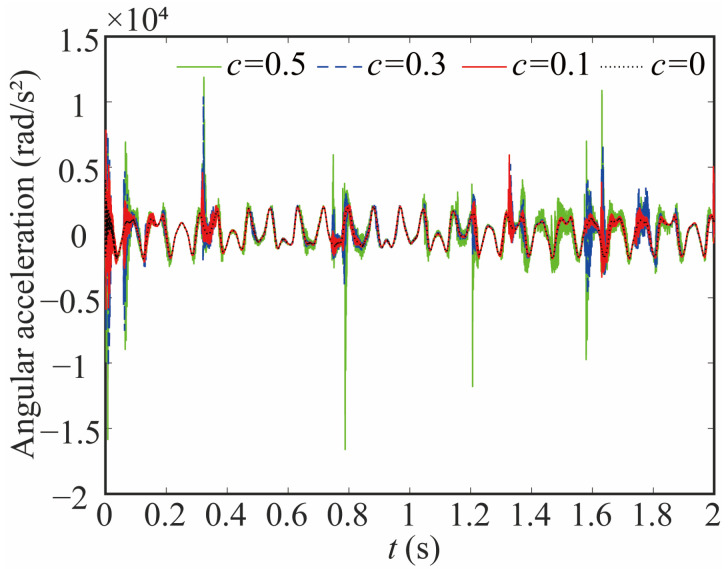
Angular acceleration of joint 1 with different clearance sizes.

The variation in the joint contact force exerts a discernible influence on the motion state of the joint. As shown in Figure 20, Figure 21 and Figure 22, the increase in the clearance value corresponds directly to an augmentation in both angular displacement and angular velocity, and the change between 0 and 0.8 s is not apparent. There is a significant increase after 0.8 s. As the clearance size increases, the angular acceleration of the joint and the magnitude of the acceleration peak undergo a corresponding augmentation. When *c* = 0.5, the frequency of the angular acceleration within 1.41–1.72 s increases. This observation aligns with the varying force trend and further indicates the direct correlation between changes in contact force and the stability of the joint. Larger clearance leads to poor system performance.

**Figure 23:**
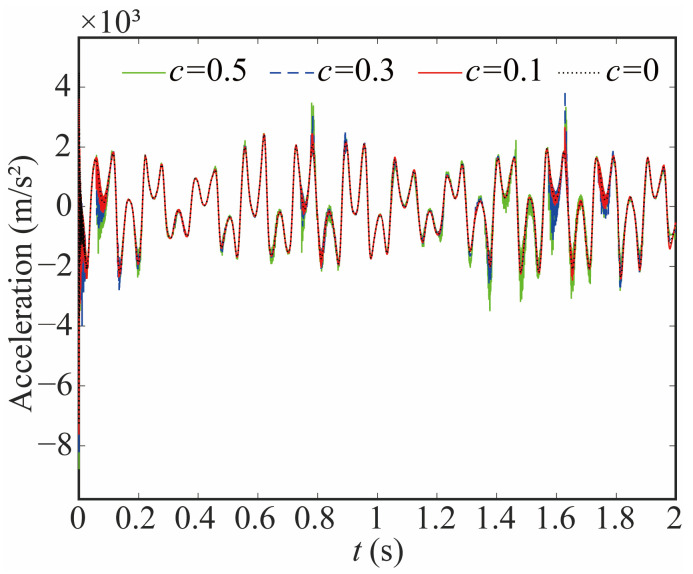
Acceleration of end-effector with different clearance sizes in X direction.

**Figure 24:**
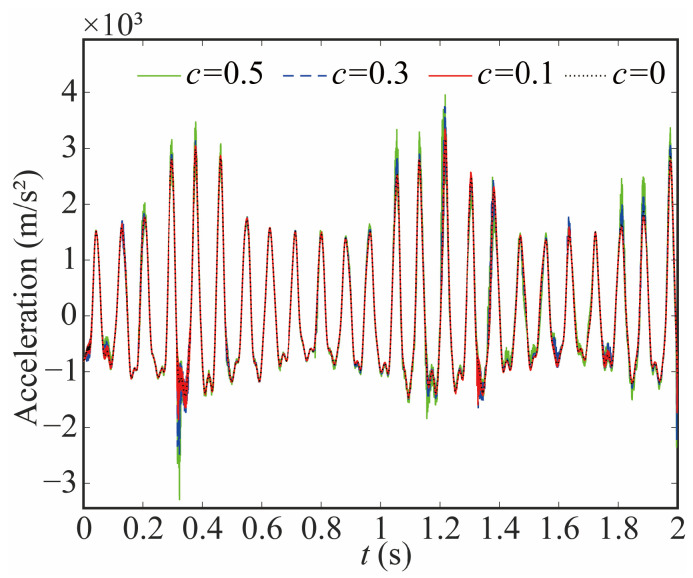
Acceleration of end-effector with different clearance sizes in Y direction.

The acceleration of the end-effector with different clearance sizes is given in Figure 23 and Figure 24. The larger joint clearance leads to a larger acceleration and a value of peak. However, compared with the acceleration of the joint, the change in the end-effector is small, and the increase in the value of peak is small. The frequency of acceleration increases, but the degree of growth is smaller than that of joint 1. The reason behind this phenomenon lies in the cumulative effect of flexible joints within the system, which effectively mitigates the acceleration impact resulting from clearance. Additionally, the flexible joint exhibits a significant damping effect on the robot, further contributing to this reduction.

## 5. Conclusions

The joint clearance and flexibility caused by the reducer are comprehensively considered to research the dynamics of the robot in this paper. The dynamic equation of the robot is derived by applying the Newton–Euler method combined with the Spong model, Coulomb friction model, and L-N model. The effects of clearance and flexible joints on the system are analyzed through numerical simulation.

The presence of clearance enhances the degree of freedom of the system, thereby introducing unpredictability in its movement. Numerical simulations reveal a notable fluctuation in the contact force within the clearance. In comparison to an ideal-joint robot, the angular acceleration of the joint and the acceleration of the end-effector exhibit an increase, and the angular acceleration and acceleration of the end-effector are greatly affected. The contact force increases with increasing clearance size, the frequency of the force also increases, and the system uncertainty becomes more significant. In addition, the amplitude and frequency of the angular acceleration and the acceleration of end also increase, which results in greater noise and lowers the reliability.

The dynamic characteristics undergo alterations when considering the flexibility of the robot joint. Compared with the rigid system, the dynamic behaviors of the flexible-joint robot all decrease to an extent and have a lag, in which the accelerations are obviously affected. This demonstrates the significant influence that joint flexibility exerts on the robot performance. Specifically, the flexible joint acts as a damping mechanism in the system, having a profound impact on the precision analysis and vibration control of the robot. At present, the research in this paper takes into account the flexibility of the robot joints and the clearances existing in the joints, but compared with the actual robot, the degree of freedom of the robot is simplified and has not been extended to the three-dimensional space. In addition, the model in this paper is mainly aimed at series robot modeling and is not applicable to parallel robots, but the modeling ideas can provide reference, and the inclusiveness and extensibility of the model in this paper need to be developed. In the future, further research will be conducted based on the proposed model. This work could be extended to three-dimensional space.

## Figures and Tables

**Table 1:** Simulation parameters.

Description	Value	Unit
Length of link 1	0.61	m
Length of link 2	0.66	m
Density	2830	kg/m^3^
Restitution coefficient	0.46	-
Friction coefficient	0.01	-
Poisson’s ratio	0.33	-
Young’s modulus	7.17 × 10^10^	N/m^2^
Nonlinear index	1.5	-
Torsional stiffness of spring 1	200	Nm/rad
Torsional stiffness of spring 2	200	Nm/rad

**Table 2:** The contact force at the peak.

t/s	0.06-	0.32-	0.74-	0.78-	1.05-	1.21-
$F_{c}/N$ (c=0.5 mm)	69,649.14	67,131.48	47,781.60	73,830.14	34,785.59	72,705.91
$F_{c}/N$ (c=0.3 mm)	27,106.01	47,742.36	24,968.80	30,575.93	26,351.63	34,631.73
$F_{c}/N$ (c=0.1 mm)	23,114.30	16,496.32	12,556.42	14,103.36	19,974.02	26,565.43
t/s	1.37-	1.48-	1.58-	1.63-	1.81-	1.97-
$F_{c}/N$ (c=0.5 mm)	49,662.00	35,175.79	58,652.83	65,669.90	35,513.15	28,366.82
$F_{c}/N$ (c=0.3 mm)	37,232.58	14,555.25	37,532.75	50,338.52	29,881.80	23,187.64
$F_{c}/N$ (c=0.1 mm)	20,992.97	13,598.44	9204.30	25,292.24	17,553.99	20,334.22

## Data Availability

The data that support the findings of this study are available from the corresponding author upon reasonable request.
